# Effect of *Clostridium butyricum* and Butyrate on Intestinal Barrier Functions: Study of a Rat Model of Severe Acute Pancreatitis With Intra-Abdominal Hypertension

**DOI:** 10.3389/fphys.2020.561061

**Published:** 2020-10-29

**Authors:** Han-bing Zhao, Lin Jia, Qing-qing Yan, Qi Deng, Bo Wei

**Affiliations:** ^1^Department of Gastroenterology, Guangzhou First People’s Hospital, Guangzhou Medical University, Guangzhou, China; ^2^Department of Clinical Medicine, Guizhou Medical University, Guiyang, China; ^3^Department of Gastroenterology, Guangzhou First People’s Hospital, South China University of Technology, Guangzhou, China

**Keywords:** severe acute pancreatitis, intra-abdominal hypertension, *Clostridium butyricum*, butyrate, intestinal barrier function

## Abstract

**Background/Aims:**

Severe acute pancreatitis (SAP) is associated with intra-abdominal hypertension (IAH) and abdominal compartment syndrome (ACS), but treatment of these conditions is difficult. We studied a rat model of SAP + IAH to determine the effect of oral administration of *Clostridium butyricum* and butyrate (its major metabolite) on intestinal barrier functions.

**Methods:**

A total of 48 rats were assigned to four groups, with 12 rats per group: Sham, SAP+IAH, SAP+IAH+*C. butyricum*, and SAP + IAH + butyrate. SAP was induced by sodium taurocholate infusion into the biliopancreatic duct, intra-abdominal pressure (IAP), mortality was measured 24 h later, and then rats were euthanized. The plasma levels of several markers [amylase, diamine oxidase (DAO), fluorescein isothiocyanate (FITC)-dextran, tumor necrosis factor alpha (TNF-α), interleukin (IL)-6, IL-1β, IL-12, lipopolysaccharide (LPS)] and fecal butyric acid level were determined. The pancreas and intestine were examined using histology, and RT-PCR and Western blotting of intestinal tissues were used to measure the expression of six markers {tight junction proteins [zonula occludens protein-1 (ZO-1), claudin-1, claudin-2, occluding], matrix metalloproteinase 9 [MMP9], and TNF-α}. The gut flora of the rats was examined by 16S rRNA sequencing.

**Results:**

Induction of SAP + IAH altered several functions of the intestinal barrier, and enhanced intestinal permeability, decreased the levels of ZO-1, claudin-1, occludin, the richness and diversity of the microflora community, the relative abundance (RA) of Firmicutes, and the number of probiotic organisms. However, induction of SAP+IAH increased the expression of claudin-2, MMP9, and TNF-α, and the RA of Proteobacteria and pathogens in the feces. Rats that received oral *C. butyricum* or butyrate had reduced intestinal injury and plasma levels of DAO, LPS, and inflammatory cytokines.

**Conclusion:**

This study of rats with SAP+IAH indicated that oral dosing of *C. butyricum* or butyrate reduced intestinal injury, possibly by altering the functions of the intestinal mucosal barrier.

## Introduction

Severe acute pancreatitis (SAP), which is characterized by necrosis of pancreatic and peri-pancreatic tissues and often accompanied by multiple organ dysfunction syndrome (MODS), has a mortality rate of 10–30% (Thandassery et al., 2015; Agarwal et al., 2016). In addition, 59–78% of patients with SAP can develop intra-abdominal hypertension (IAH), which can progress to abdominal compartment syndrome (ACS), and concurrent SAP with ACS has a mortality rate as high as 66.7% (Ke et al., 2012a).

The healthy intestines of humans contain many bacteria, some of which produce endotoxins. In SAP, the release of inflammatory mediators and cytokines, ischemia and reperfusion, dysbacteriosis, and other factors disrupt the mucosal barrier of the intestine and can lead to intestinal failure (Ammori et al., 2002; Yasuda et al., 2006). Damage of the intestinal barrier is characterized by increased mucosal and vascular permeability, and this can cause translocation of pathogens and endotoxins from the intestinal tract to the regional lymph nodes, portal veins, and peripheral blood system, leading to systemic endotoxemia. These pathogens and endotoxins can also promote pancreatic necrosis, increase the release of proinflammatory factors, and ultimately cause multiple organ failure (MOF).

Previous research has confirmed that increased intra-abdominal pressure (IAP) correlates with bacterial translocation and intra-abdominal infection, and that ACS is related to pancreatic necrosis and infection (Gong et al., 2009; Ke et al., 2012b). Our previous study (Li et al., 2013) indicated that the IAP in the rat model of SAP increased rapidly as disease progressed, and that changes in the intestinal barrier of rats with SAP and IAH were especially obvious at 6 h after induction. This indicates that treatment of SAP with IAH must be received within an early therapeutic window to maintain intestinal barrier (Li et al., 2013). Clinicians have examined various potential treatments to maintain the intestinal barrier function, alleviate enterogenic infection, and alleviate SAP. There is an urgent need for new treatments or drugs that protect the intestinal barrier. One proposed strategy is probiotic therapy.

Previous studies have reported that probiotics can alleviate the symptoms of many intestinal diseases, such as acute and chronic diarrhea, inflammatory bowel disease, and irritable bowel syndrome, because of their ability to regulate the balance of intestinal flora, maintain the intestinal barrier function, regulate intestinal immunity, and inhibit the generation of inflammatory factors (Sanders et al., 2013). Moreover, a previous study documented that administration of probiotic therapy to rats can inhibit the development of sepsis (Liu et al., 2013). In this context, it must be considered that SAP with IAH can exacerbate intestinal barrier functional disturbance (IBFD). Thus, in theory, probiotics could be used as a simple, effective, and comprehensive treatment to maintain intestinal barrier function in the presence of SAP with IAH. However, there is still no consensus regarding the use of probiotic therapy for treatment of SAP because the limited clinical trials have produced disparate results (Bongaerts and Severijnen, 2016). Thus, current clinical practice guidelines only rarely recommend the use of probiotics. Some researchers believe the disparate clinical results are due to the use of different types of probiotics and treatment protocols. Thus, it is possible that patients with SAP may benefit from a probiotic regimen if the appropriate doses and treatment durations are used (Gou et al., 2014).

At present, very few *in vivo* studies have examined changes in the intestinal barrier and barrier-associated microbiota in the presence of SAP with IAH. The general purpose of this study is to identify probiotics that regulate the intestinal barrier function in the presence of SAP with IAH and to determine the mechanism of this effect.

*Clostridium butyricum* is an anaerobic bacterium in the intestine that can ferment dietary fibers to produce short-chain fatty acids (SCFAs), mainly acetic acid, propionic acid, and butyric acid. Several studies have shown that *C. butyricum* and butyrate can help to maintain intestinal micro-ecology and immune modulation, attenuate the activation of inflammatory pathways, and maintain the stability of the intestinal barrier in the presence of intestinal inflammatory diseases (Corridoni et al., 2012; Miao et al., 2016). However, the effect and mechanism of *C. butyricum* and butyrate on protecting the intestinal mucosal barrier in the presence of SAP with IAH are unknown.

The specific aims of this study of a rat model of SAP were to observe changes of the intestinal mucosal barrier and the flora after induction of SAP with IAH, and to examine the protective effect of oral administration of *C. butyricum* or butyrate on the mechanical, capillary, endothelial, and biological functions of the intestinal barrier. These findings may provide a basis for the development of clinical treatments for SAP with IAH.

## Materials and Methods

### Experimental Animals

All experimental procedures were performed in compliance with the guidelines of the Institutional Animal Care and Use Committee. This study was approved by the Animal Ethics Committee of School of Medicine, South China University of Technology. Forty-eight specific pathogen-free (SPF) female Sprague–Dawley rats weighing 244 ± 15 g (mean ± SD) were purchased from the Experimental Animal Center of Southern Medical University (license number SCXK 2016-0041) and raised in an SPF animal room with a 12-h light/dark cycle.

After 1 week of adaptive feeding, rats were randomly allocated into one of four groups (12 rats per group): sham operated (Sham), SAP with IAH alone (SAP + IAH), SAP with IAH and *C. butyricum* treatment (*C. butyricum*), and SAP with IAH and sodium butyrate treatment (Butyrate). Rats in the *C. butyricum* Group were given 1 × 109 colony-forming units (CFUs) of *C. butyricum*, and those in the Butyrate Group were given 100 mg/kg body weight of sodium butyrate (Meilun Biotechnology co., Ltd., Dalian, China) in 1.0 ml of normal saline; rats in the Sham Group and SAP + IAH Group were given 1.0 ml of normal saline alone. These treatments were administered by oral gavage once a day for 10 days. On day 11, after the rats were resuscitated from anesthesia used for induction of SAP, each group was given one additional treatment.

### Culture of *Clostridium butyricum*

*Clostridium butyricum* (ATCC 19398, Beijing Kexing Biotechnology Co., Ltd., Beijing, China) was cultured anaerobically at 37°C for 24 h in a medium (pH 6.8) consisting of 10 g tryptone, 10 g beef extract, 5 g glucose, 5 g sodium chloride, 3 g yeast extract, 3 g sodium acetate, 1 g soluble starch, 0.5 g L-cysteine hydrochloride, and 15 g agar. Bacteria were harvested and washed 3 times with ice-cold PBS, and then suspended in saline to obtain a bacterial suspension.

### Severe Acute Pancreatitis Induction

Before induction of SAP, rats were deprived of food, but not water, for 12 h. SAP was induced using the modified Aho method (Aho et al., 1980; Jha et al., 2008). In brief, sodium pentobarbital (40 mg/kg) was given for abdominal cavity anesthesia, and all efforts were made to minimize pain. Along the duodenal wall, the debouchment of the biliopancreatic duct was identified and then, using speed-controlled (0.1 ml/min) retrograde infusion, 4.5% sodium taurocholate (0.1 ml/100 g; Meilun Biotechnology Co., Ltd., Dalian, China) was introduced into the biliopancreatic duct. Rats in the sham group received normal saline (0.1 ml/100 g) alone. At the end of surgery, normal saline (20 ml/kg) was subcutaneously injected into the back of rats in each group to supplement the loss of fluid during surgery. At 24 h after the operation, the IAP of each rat was determined. Rats were then sacrificed with an overdose of sodium pentobarbital(160 mg/kg) given for abdominal cavity injection to minimize pain, and samples of whole blood, pancreatic tissue, the distal 10 cm of the ileum, and feces in the distal ileum were collected.

### Determination of Intra-Abdominal Pressure

The BL-420F Biological Function Experimental System (Chengdu TME Technology Co., Ltd., Chengdu, China) was used to measure IAP. This instrument consists of a biological information collector, a high-precision pressure transducer (pressure range: -8 to 50 mmHg, accurate to within 0.1 mmHg), a pressure measuring tube, a functional experiment system software, and a computer. IAP was measured after anesthesia at 24 h after surgery, while the rats were supine. The pressure measuring tube was inserted through the abdomen wall, and into the lower left quadrant, with guidance by a 20 gauge needle that was connected to the high-precision pressure transducer. The system software in the computer provided the IAP data.

### Gas Chromatography Analysis of Fecal Butyric Acid Content

Feces in the distal ileum were collected at 24 h after induction of SAP and then stored at -80°C for later analysis. Concentrations were measured by 6890N Network gas chromatography System (Agilent Technologies Co., Ltd., United States) as previously described (Scheppach et al., 1987). Briefly, feces (0.1 g) were first emulsified in 500 μl of saturated NaCl solution. Thereafter, 40 μl of 10% sulfuric acid was added to acidify the fecal samples. Next, SCFAs were extracted from the samples with 1 ml of diethyl ether. Samples were then centrifuged at 14,000 r/min for 15 min at 4°C. The supernatants were used for analysis with gas chromatography.

### Histopathologic Examination

Tissues from the pancreas and distal ileum were fixed in 10% formalin, embedded in paraffin, and 4 μm sections were stained with hematoxylin and eosin (H&E). The pathological changes of pancreas and ileum were evaluated by two professional pathologists who were blinded to group allocations, using the criteria of Chiu et al. (1970) and Schmidt et al. (1992).

### Intestinal Permeability *in vivo*

Intestinal permeability *in vivo* was measured as described previously with some modifications (Cani et al., 2008). Rats were fasted for 4 h before the end of the experiment and then orally given fluorescein isothiocyanate (FITC)-dextran (4 kDa; Sigma–Aldrich Co., Ltd., St. Louis, MO, United States) in saline (600 mg⋅kg^–1^, 125 mg/ml). After 4 h, rats were sacrificed with an overdose of sodium pentobarbital. Plasma was isolated using centrifugation, and plasma FITC-dextran levels were analyzed with a fluorescence spectrophotometer (F-7000, HITACHI, Japan).

### Determination of Plasma Biochemical Indicators

After measurement of IAP, blood samples were collected from the abdominal aorta. An automatic analyzer (AU5400; Olympus, Tokyo, Japan) was used to determine amylase activity. The levels of diamine oxidase (DAO), tumor necrosis factor alpha (TNF-α), interleukin (IL)-6, IL-1β, and IL-12 were detected using the enzyme-linked immunosorbent assay (ELISA) kit (Elabscience Biotechnology Co., Ltd., Wuhan, China). Plasma lipopolysaccharide (LPS) was measured using the chromogenic Limulus amebocyte lysate assay (XIAMEN BIOENDO Technology Co., Ltd., Xiamen, China).

### Reverse Transcription-Polymerase Chain Reaction

The mRNA levels of zonula occludens protein-1 (ZO-1), claudin-1, claudin-2, occludin, and matrix metalloproteinase 9 (MMP9) were determined using the reverse transcription-polymerase chain reaction (RT-PCR). The TRIzol reagent (Invitrogen, Technology Co. Ltd., CA, United States) was used to extract total RNA from the distal ileum at 24 h after induction of SAP, according to the instruction manual. To amplify complementary DNA fragments, isoform-specific quantitative PCR (qPCR) primers were used (Table 1). RT-PCR was performed using the PrimeScript^TM^ RT reagent Kit with gDNA Eraser, and qPCR was performed using the SYBR^®^ Premix Ex Taq^TM^ II (Tli RNaseH plus) kit (TAKARA, Biotechnology Co., Ltd., Japan). The 2^–ΔΔ*Ct*^ method was used to calculate changes in gene expression relative to β-actin.

**TABLE 1:** Polymerase chain reaction (PCR) primers (5′–3′) used for analysis of the distal ileum.

**Gene**	**Forward**	**Reverse**
ZO-1	GAGTCTAGCAGCTGGACTAAG	GAACCTCTAGCCAATGCCTG
Claudin-1	GCTAAGCTGCTAACCCTGTG	GCAAGTCTAGCAGTTTGTGTAG
Claudin-2	CGAGAAAGAACAGCTCCGTTT	TTCGCTTGTCTTTTGGCTGC
Occludin	CAGAACTCGACGAGGTCAAC	CAGGTAGCTAGATGCACCTC
MMP9	GCAGCGCAAGCCTCTAGAG	GCTGATTGGTTCGAGTAGCTG
β-Actin	CACCCGCGAGTACAACCTTC	CCCATACCCACCATCACACC

### Western Blot Analysis

Ileum tissues were lyzed with RIPA buffer (KW, Biotechnology Co., Ltd., Beijing, China) for 30 min on ice and then centrifuged at 12,000 r/min for 15 min at 4°C. Supernatants were harvested, and protein content was determined using the BCA method (KW, Biotechnology Co., Ltd., Beijing, China). Western blot analyses were performed according to standard protocols. Samples (40 μg protein) were subjected to tris-glycine sodium dodecyl sulfate-polyacrylamide gel electrophoresis (SDS-PAGE), and then electrotransferred to a polyvinylidene difluoride (PVDF) membrane (Millipore, MA, United States). Membranes were blocked using a Tris-buffer saline solution containing 0.05% Tween 20 (TBS-T) and 5% non-fat dry milk at room temperature for 1 h. The membranes were then incubated with the following primary antibodies: rabbit antibody to ZO-1, claudin 1, claudin-2, occludin, MMP9, TNF-α, and β-actin (1:1,000; Abcam, Cambridge, United Kingdom) for 1 h at room temperature under slight agitation, followed by incubation at 4°C overnight. The membranes were then washed for 10 min with TBS-T three times, and the secondary antibody [horseradish peroxidase (HRP)-conjugated goat anti-rabbit IgG antibody; 1:6,000; TDY, Biotechnology Co., Ltd., Beijing, China] was added for 1 h at room temperature. The ECL substrate was added, and the images were obtained using the Bio-Rad Gel Doc EZ gel imaging system. Gray-scale values of the target bands were quantified using Image J software (NIH).

### Community Diversity of Intestinal Microflora (Feces in the Distal Ileum Were Collected From Five Randomly Selected Rats per Group for rRNA Sequencing)

#### DNA Extraction and PCR Amplification

Microbial DNA was extracted using the E.Z.N.A. stool DNA kit (Omega Bio-tek, Norcross, GA, United States) according to the manual guidelines. The V4 region of bacterial 16S rRNA genes was amplified by PCR (95°C for 2 min; 25 cycles at 95°C for 30 s, 55°C for 30 s, 72°C for 30 s; and a final extension at 72°C for 5 min) using the primers 515F (5′-GTGCCAGCMGCCGCGGTAA-3′) and 806R (5′-GGACTACHVGGGTWTCTAAT-3′). All PCR reactions were performed in triplicate in a 25-μl reaction mixture containing 2 μl of DNTPs (2.5 mmol/L), 0.2 μl of HotStart Taq DAN Polymerase, 0.2 μl of each primer (20 pmol/μl), 1 μl of template DNA, 2.5 μl of 10× buffer, and 19.1 μl of ddH_2_O.

#### Illumina Hiseq 2500 Sequencing

Amplicons were extracted from 2% agarose gels and purified using the QIAquickDNA Gel Extraction kit (TIANGEN, Biotechnology Co., Ltd., Beijing, China) according to the manual guidelines. Quantification of amplicons was then performed using QuantiFluor-ST (Promega, United States). Next, the amplicons were pooled in equimolar quantities and analyzed using paired-end sequencing (2 × 250) on an Illumina Hiseq 2500 platform with standard protocols.

#### Processing of Sequencing Data and Data Analysis

All raw “fastq” files were subjected to demultiplexing and quality filtering using QIIME (version 1.80). In this procedure, reads with 250 bp that were truncated at any site were assigned an average quality score below 20 over a 10-bp sliding window, and all reads shorter than 50 bp were excluded. Then barcode matching was performed, in which reads with two nucleotide mismatches in primer matching and those with ambiguous characters were excluded. Finally, sequences with overlaps greater than 10 bp were assembled. Any read that could not be assembled was excluded.

UPARSE (version 7.1^1^) was used to characterize sequences with 97% identity or more, and these sequences were considered to be in the same operational taxonomic unit (OTU). UCHIME (version 4.2.40) was used to identify and remove all chimeric sequences. Phylogenetic relationships were determined from 16S rRNA gene sequences using the RDP Classifier (version 2.2) and the SILVA 16S rRNA database (SSU115, Max Planck Institute, Germany), with a confidence threshold of 70%. QIIME was used to plot rarefaction curves and to measure alpha diversity (Chao and Shannon indices) and beta diversity. The OTUs for each sample were used for unweighted UniFrac distance-metrics analysis. Then, principal component analysis (PCA) was performed based on matrix-of-distance. The Mann–Whitney test was used at the levels of phyla and genera to determine the impact of SAP + IAH on microfloral diversity.

### Statistical Analysis

SPSS 16.0 was used for statistical analysis. Data are expressed as means ± standard deviations (SDs) and a one-way analysis of variance (ANOVA) and the least significant difference *t-*test (LSD *t*-test) or the Kruskal–Wallis test were used to determine the significance of differences for multiple comparisons among groups. A *P*-value below 0.05 was considered significant.

## Results

### Levels of IAP, Mortality, and Fecal Butyric Acid Level After Induction of Severe Acute Pancreatitis

**FIGURE 1:**
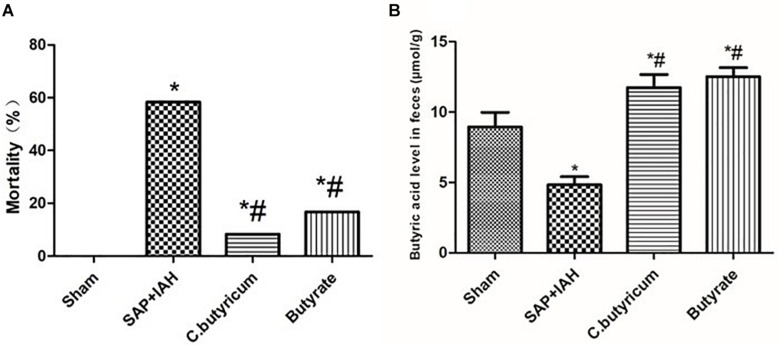
The mortality and fecal butyric acid level of different treatments. **(A)** Mortality. **(B)** Fecal butyric acid level at 24 h after induction of severe acute pancreatitis (SAP). Here and below: data are shown as means ± SDs; *P < 0.05 *vs.* Sham Group; ^#^*P* < 0.05 *vs.* SAP + intra-abdominal hypertension (IAH) Group.

At 24 h after surgical induction of SAP, the IAP was 0.8–4.7 mmHg in the Sham Group, 12.8–22.7 mmHg in the SAP + IAH Group, 12.0–16.4 mmHg in the *C. butyricum* Group, and 13.1–15.9 mmHg in the Butyrate Group (data not shown). These results confirm the successful establishment of this rat model of SAP with IAH. The mortality of the *C. butyricum* Group and the Butyrate Group was significantly lower than that of the SAP + IAH Group at the end of the experiment (*P* < 0.05) (Figure 1A). However, the levels of fecal butyric acid in the two treatment groups were significantly higher than that of the SAP + IAH Group (*P* < 0.05; Figure 1B).

### Pathological Changes in the Pancreas and Small Intestine

**FIGURE 2:**
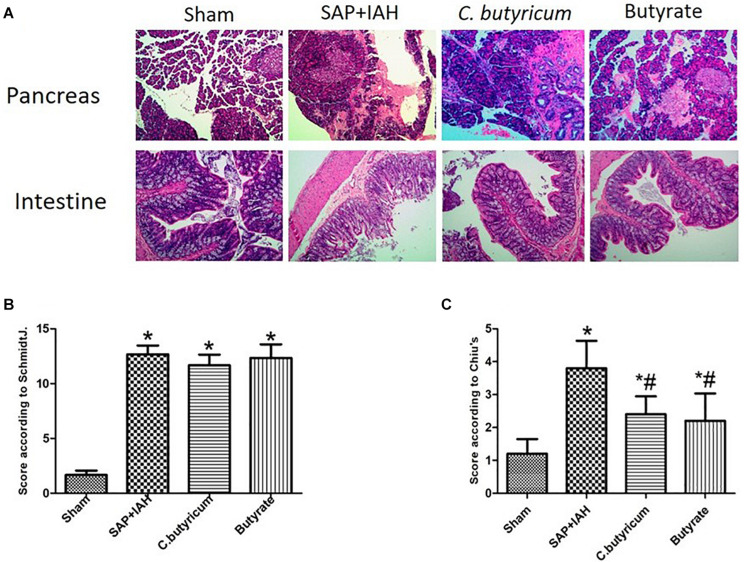
Effect of different treatments on the histopathology of the pancreas and small intestine at 24 h after induction of SAP (**A:** representative photomicrographs), pathological scores for the pancreas (**B:** Schmidt method), and pathological scores for the small intestine (**C:** Chiu method). *P < 0.05 vs. Sham Group; ^#^*P* < 0.05 vs. SAP+IAH Group.

Pancreatic tissues had no significant changes in the Sham Group, but there were obvious histopathological changes (acinar cell necrosis, hemorrhage, and inflammatory cell infiltration) in the other three groups (Figure 2A). In agreement, the pathological scores were significantly lower in the Sham Group than in the other three groups (*P* < 0.05), but the scores in the two treatment groups were not significantly different from that of the SAP + IAH Group (*P* > 0.05; Figure 2B).

Intestinal tissues had no significant changes in the Sham Group. There were significant pathological changes (massive destruction of villi, mucosal erosion, and inflammatory cell infiltration) in the SAP + IAH Group, but less damage in the two treatment groups (Figure 2A). The pathological severity scores confirmed that these two treatments reduced the severity of damage, but not to the level in the Sham Group (*P* < 0.05; Figure 2C).

### Plasma Levels of Amylase, Diamine Oxidase, Lipopolysaccharide, Fluorescein Isothiocyanate-Dextran, Tumor Necrosis Factor Alpha, Interleukin-6, Interleukin-1β, and Interleukin-12

**FIGURE 3:**
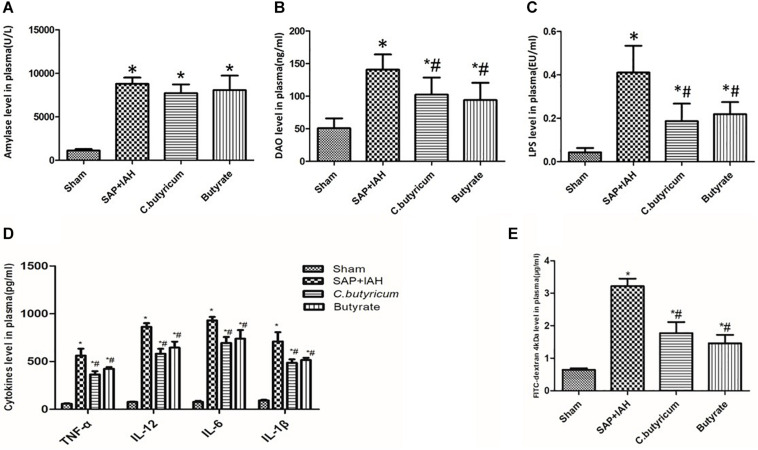
Effect of different treatments on the plasma levels of amylase **(A)**, diamine oxidase (DAO) **(B)**, lipopolysaccharide (LPS) **(C)**, tumor necrosis factor alpha (TNF-α), interleukin (IL)-6, IL-1β, and IL-12 **(D**), and fluorescein isothiocyanate (FITC)-dextran **(E)** at 24 h after induction of SAP. *P < 0.05 vs. Sham Group; ^#^*P* < 0.05 vs. SAP+IAH Group.

The amylase levels in the SAP + IAH Group and the two treatment groups were significantly higher than that of the Sham Group (*P* < 0.05). Although the amylase levels in the two treatment groups were lower than in the SAP + IAH Group, these differences were not significant (*P* > 0.05; Figure 3A). The plasma DAO, LPS, FITC-dextran, and inflammatory cytokines levels of the SAP + IAH Group and the two treatment groups were significantly higher than that of the Sham Group (*P* < 0.05). In addition, the DAO, LPS, FITC-dextran, and inflammatory cytokine levels of the two treatment groups were similar to each other (*P* > 0.05) and significantly lower than that of the SAP + IAH Group (*P* < 0.05; Figures 3B–E).

### Expression of Zonula Occludens Protein-1, Claudin-1, Claudin-2, Occludin, and Matrix Metalloproteinase 9 mRNAs in the Small Intestine

**FIGURE 4:**
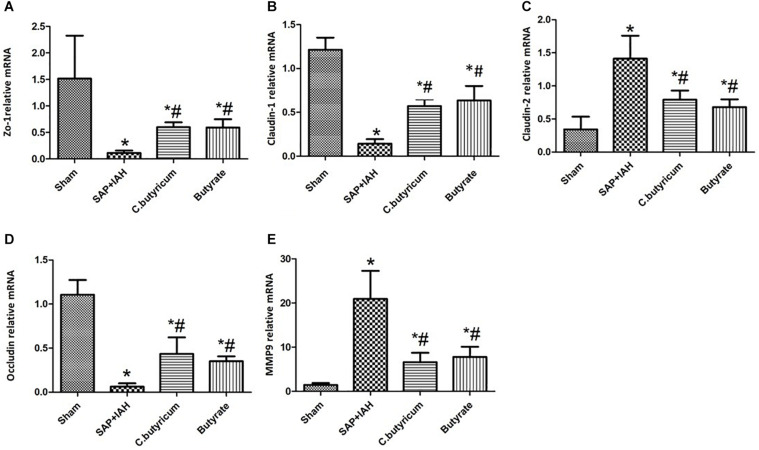
Effect of different treatments on the mRNA levels of zona occludens protein 1 (ZO-1) **(A)**, claudin-1 **(B)**, claudin-2 **(C)**, occludin **(D)**, and matrix metalloproteinase (MMP9) **(E)**, in intestinal tissues at 24 h after induction of SAP. *P < 0.05 vs. Sham Group; ^#^*P* < 0.05 vs. SAP+IAH Group.

Analysis of the expression of mRNAs of three TJ proteins (ZO-1, claudin-1, and occludin) indicated greater expression in the Sham Group than in the three other groups (*P* < 0.05), and greater expression in the two treatment groups than in the SAP + IAH Group (*P* < 0.05; Figures 4A,B,D). In addition, expression of claudin-2, MMP9 was lower in the Sham Group than in the three other groups (*P* < 0.05) and lower in the two treatment groups than in the SAP + IAH Group (*P* < 0.05; Figures 4C,E).

### Expression of Zonula Occludens Protein-1, Claudin-1, Claudin-2, Occludin, Matrix Metalloproteinase 9, and Tumor Necrosis Factor Alpha Proteins in the Small Intestine

**FIGURE 5:**
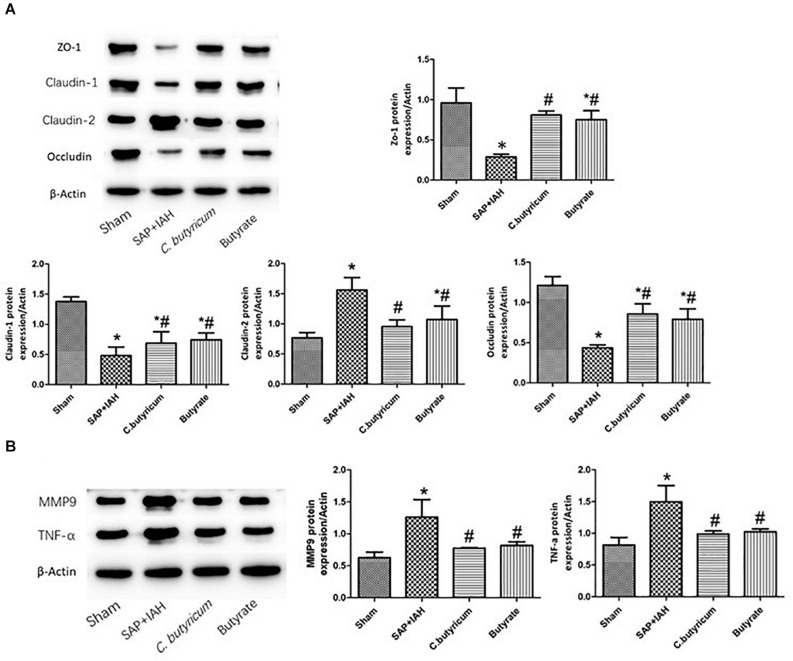
Effect of different treatments on the protein levels of ZO-1, claudin-1, claudin-2, and occludin **(A)**, MMP9 and TNF-α **(B)**, in intestinal tissues at 24 h after induction of SAP. *P < 0.05 vs. Sham Group; ^#^*P* < 0.05 vs. SAP+IAH Group.

Western blotting analysis of the same three TJ proteins (ZO-1, claudin-1, and occludin) indicated the same trends for their mRNAs (i.e., high levels in the Sham Group, intermediate levels in the two treatment groups, and low levels in the SAP + IAH Group; *P* < 0.05; Figure 5A). In addition, relative to the Sham Group, the levels of claudin-2, MMP9, and TNF-α were significantly higher in the SAP + IAH Group (*P* < 0.05); relative to the SAP + IAH group, the levels of claudin-2, MMP9, and TNF-α were significantly lower in the two treatment groups (*P* < 0.05; Figures 5A,B).

### MiSeq Sequencing Results, Chao and Shannon Indices, Principal Component Analysis

**FIGURE 6:**
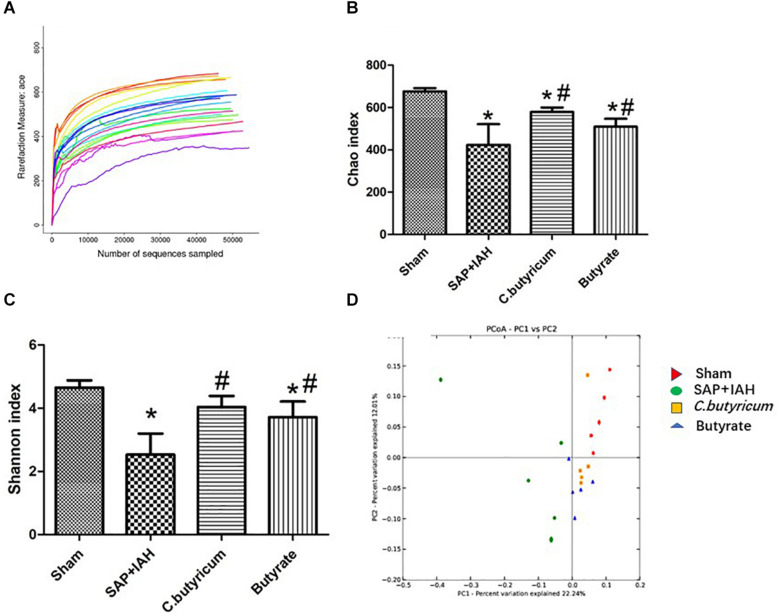
Rarefaction curves of different samples **(A)**, effect of different treatments on the Chao index **(B)**, and Shannon index **(C)**, and principal component analysis (PCA) plots **(D)**, of fecal microflora at 24 h after induction of SAP. *P < 0.05 vs. Sham Group; ^#^*P* < 0.05 vs. SAP+IAH Group.

We obtained an average of 61,615 valid reads from 20 samples by MiSeq sequencing (five feces samples from the distal ileum of each group). The rarefaction curves approached saturation, indicating the sequencing was deep enough to capture most of the OTUs in our samples (Figure 6A). In addition, relative to the Sham Group, the Chao and Shannon indices were significantly lower in the SAP + IAH Group (*P* < 0.05); relative to the SAP + IAH group, the Chao and Shannon indices were significantly higher in the two treatment groups (*P* < 0.05, Figures 6B,C). This demonstrates that SAP + IAH decreased microflora community richness and diversity, but treatment with *C. butyricum* or butyrate significantly increased richness and diversity. The PCA showed significant differences in the microfloral structure of feces in the distal ileum among the Sham group, the SAP + IAH group, and the two treatment groups, although the two treatment groups were similar (Figure 6D).

### Changes in the Microbial Composition

**FIGURE 7:**
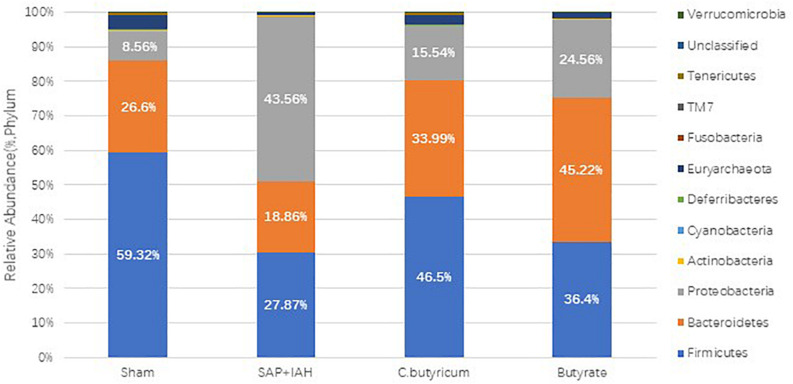
Effect of different treatments on the microbial composition of feces at the phylum level at 24 h after induction of SAP.

The microbial composition was quite different in the SAP + IAH experimental group, Sham group, and the two treatment groups (Figure 7). At the phylum level, the microflora community of the distal ileum in all four groups was dominated by Firmicutes, Bacteroidetes, and Proteobacteria, although the relative abundance (RA) of these phyla differed among groups. The SAP + IAH group had a predominance of Firmicutes and Proteobacteria, but a decreased RA of Firmicutes (SAP + IAH *vs*. Sham: 27.87 *vs*. 59.32%, *P* < 0.05) and an increased RA of Proteobacteria (SAP + IAH *vs*. Sham: 43.56 *vs*.8.56%, *P* < 0.05). However, the *C. butyricum* and butyrate groups had a significantly increased RA of Firmicutes and a decreased RA of Proteobacteria (*P* < 0.05). In addition, the RAs of Firmicutes and Proteobacteria were similar in the two treatment groups. The RA of Bacteroidetes was not different among the four groups.

**FIGURE 8:**
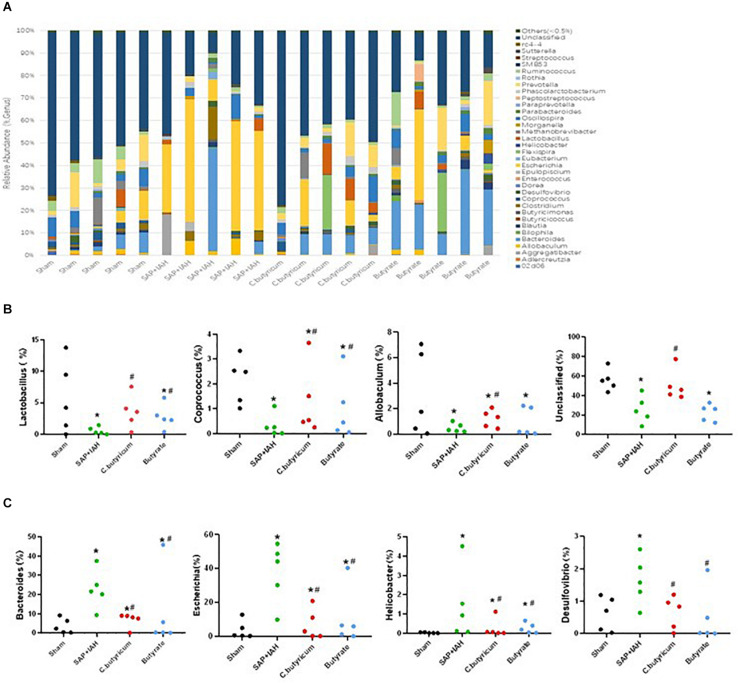
Effect of different treatments on the microbial composition of feces at the genus level at 24 h after induction of SAP. **(A)** Distribution of bacterial taxa. **(B,C)** Relative abundance of operational taxonomic units (OTUs). Sequences that could not be classified into any known group were designated as “Unclassified”. *P < 0.05 vs. Sham Group; ^#^*P* < 0.05 vs. SAP+IAH Group.

We also examined the influence of IAH on the RA of intestinal microflora at the genus level (Figure 8A), by analysis of the impact of different treatments on the 37 most abundant microflora genera. The numbers of *Lactobacillus*, *Coprococcus*, and *Allobaculum* were significantly lower in the SAP + IAH Group than in the Sham Group (*P* < 0.05), but treatment with *C. butyricum* or butyrate significantly increased the numbers of the three probiotics (*P* < 0.05; Figure 8B). In addition, the numbers of *Bacteroides*, *Escherichia*, *Helicobacter*, and *Desulfovibrio* were significantly greater in the SAP + IAH Group than in the Sham Group, but treatment with *C. butyricum* or butyrate significantly decreased the numbers of these four pathogenic species (*P* < 0.05; Figure 8C).

## Discussion

Severe acute pancreatitis is a common and serious emergency in the clinic, and there is a high incidence of IAH in patients with SAP. This is a serious life-threatening complication, especially when ACS develops during the course of SAP (Ke et al., 2012a). In the present study, we established a rat model of SAP using speed-controlled retrograde injection of sodium taurocholate into the pancreatic duct and measured the IAP of rats in each group at 24 h after surgery. The IAP of the Sham Group ranged from 0.8 to 4.7 mmHg, but the IAPs of the other three groups were all 12 mmHg or more. The World Society of Abdominal Compartment Syndrome (WSACS) defines IAH in humans as a sustained or repeated pathological elevation of IAP to at least 12 mmHg (Kirkpatrick et al., 2013) and a normal IAP in humans as approximately 0–5 mmHg. We therefore infer that we successfully induced IAH in our rat model. In agreement, the elevation of plasma amylase and the obvious histological damage to the pancreas support the successful establishment of our rat model of SAP with IAH.

Severe acute pancreatitis complicated by IAH/ACS increases the risk for sepsis (Liao et al., 2018). The human intestine contains many microorganisms, and some of these Gram-negative bacteria can produce the toxin LPS. Our results indicate that SAP with IAH increased intestinal mucosal and vascular permeability to LPS and bacteria. This could lead to infection or necrosis of the pancreas, and even systemic inflammatory response syndrome (SIRS) and MODS. There are still no known effective clinical treatments for intestinal injury due to SAP complicated by IAH/ACS. The present study of a rat model suggests that treatment with *C. butyricum* or butyrate may attenuate intestinal injury induced by SAP and IAH, and thereby protect the intestinal barrier function and reduce mortality at the end of the experiment.

The probiotic *C. butyricum* is a Gram-positive, rod-shaped anaerobe that can ferment undigested dietary carbohydrates in the human gut to SCFAs, especially butyric acid. Oral administration of *C. butyricum* M588 leads to high production of butyrate and protects against dextran sodium sulfate-induced colitis in rats (Okamoto et al., 2000). Butyrate provides most of the energy for intestinal epithelial cells and also has anti-inflammatory and antiapoptotic effects (Ichikawa et al., 2002). A previous study showed that dietary supplementation with butyrate protected against acetic acid-induced colitis in piglets (Hou et al., 2014). Qiao et al. (2015) observed that butyrate significantly ameliorated intestinal ischemia and reperfusion injury *via* regulation of TJ proteins and inhibition of the infiltration of inflammatory cells into the intestinal mucosa. Another study reported that SCFAs, especially butyrate and propionate, have anti-inflammatory effects due to their suppression of the secretion of MMPs into the colon (Kawamura et al., 2009). All of these results are in agreement with our results, by gas chromatography, we observed that the amount of butyric acid in the feces of the two treatment groups was higher than in the SAP + IAH Group, and suggest that *C. butyricum* and its major fermentation product (butyrate) provide significant benefits by maintaining normal intestinal morphology and ameliorating colitis.

We found that induction of SAP with IAH led to significant pathological changes in the intestinal mucosa, with massive destruction of the villi, mucosal erosion, and inflammatory cell infiltration. The mucosal damage indicated an injury of the intestinal barrier function and increased mucosal permeability to LPS which is a key to causing further aggravation of inflammation (Uhl et al., 1999), based on our finding of increased levels of plasma LPS and inflammatory cytokines after induction of SAP with IAH. Notably, oral administration of *C. butyricum* or butyrate alleviated this damage and also reduced the plasma level of LPS and inflammatory cytokines.

A healthy intestinal mucosal mechanical barrier consists of intact epithelial cells and paracellular junction complexes, including TJ proteins. This barrier prevents the movement of bacteria, LPS, and other harmful substances from the intestinal lumen into the bloodstream. The major TJ proteins are claudin-1, occludin (transmembrane proteins), and ZO-1 (a peripheral membrane protein), which strengthen the intestinal barrier to decrease paracellular permeability, while the pore-forming claudin 2 protein leads to increased paracellular permeability (Turner, 2009). Upon damage of intestinal epithelial cells, DAO (secreted by intestinal cells) moves from the cytoplasm into the blood circulation (Wu and Li, 1999). In agreement, we found that the SAP + IAH Group had an elevated plasma level of DAO, intestinal tissues had underexpressed mRNA and protein levels of ZO-1, claudin-1, and occludin, while overexpressed claudin-2, and that treatment with *C. butyricum* or butyrate partially reversed these pathological responses.

We also investigated the intestinal capillary barrier by measuring the expression of MMP9. Proteins in the MMP family are calcium-dependent neutral proteases that play important roles in degrading the capillary basement membrane, which may increase microvascular permeability (Tyagi et al., 2005). A previous study showed that stimulation by several mediators of inflammation led to significant upregulation of MMP9 (Pan et al., 2017). Increased capillary leakage of the intestine can cause enteric edema and high production of bloody ascites, ultimately exacerbating IAH. TNF-α is a proinflammatory cytokine that contributes to inflammation during SAP (Duell et al., 2006). In the present study, rats with SAP + IAH had increased mRNA and protein levels of MMP9 and TNF-α, the level of TNF-α was closely linked with the expression of TJ proteins and MMP9, and oral administration of *C. butyricum* or butyrate downregulated the mRNA and protein levels of MMP9 and TNF-α.

Severe acute pancreatitis + IAH also induced dysbiosis of the intestinal microbiota in rats, as demonstrated by a decreased RA of Firmicutes, and increased levels of Proteobacteria at the distal ileum. At the genus level, we found that three probiotics (*Lactobacillus*, *Coprococcus*, and *Allobaculum*) and four pathogens (*Bacteroides*, *Escherichia*, *Helicobacter*, and *Desulfovibrio*) differed among the four groups. Probiotics are important parts of the healthy human intestinal flora, and a balance of intestinal microbial flora helps to maintain the biological barrier of the intestine. However, overgrowth of pathogens, such as *Escherichia coli*, can lead to elevated levels of LPS. Compared with the Sham Group, the SAP + IAH Group had fewer probiotics and more pathogens. In addition, treatment with *C. butyricum* or butyrate reversed this imbalance. These results are consistent with a previous finding that consumption of *C. butyricum* benefited the microbial ecosystem of the gut (Kong et al., 2011).

We also found that although the two treatment groups had lower histological scores for the pancreas and lower levels of amylase than the SAP + IAH Group, these differences were not statistically significant. Our results thus differ from the results of previous studies (Mufluoglu et al., 2006). We speculate that this may be because our direct and rapid induction of SAP caused severe damage to the pancreas, so the two treatments may not have had time to reduce the early damage to the pancreas. Moreover, because of the short time between onset of disease and collection of samples (24 h), we could not assess the effects of treatments on infectious complications in the pancreas at later phases of disease (≥24 h).

## Conclusion

Intestinal barrier functional disturbance contributes to the development of MODS and occurs early during the pathogenesis of SAP complicated by IAH. Our results suggest that the beneficial effects of *C. butyricum* and butyrate may be due to their preservation of intestinal epithelial cells, paracellular TJ proteins, the capillary basement membrane, the balance of intestinal microbial flora, and the suppression of inflammation in the intestinal mucosa and system. Our results also suggest a new direction for research and a therapeutic target for the prevention and treatment of intestinal injury caused by SAP with IAH.

## Data Availability Statement

The sequence data have been uploaded to the Sequence Read Archive (SRA) repository, accession number: PRJNA665273.

## Ethics Statement

The animal study was reviewed and approved by the Animal Ethics Committee of School of Medicine, South China University of Technology.

## Author Contributions

HZ and LJ designed the research. HZ, QY, and QD performed the research. HZ and BW analyzed the data. HZ and LJ wrote the manuscript. All authors contributed to the article and approved the submitted version.

## Conflict of Interest

The authors declare that the research was conducted in the absence of any commercial or financial relationships that could be construed as a potential conflict of interest.

## Footnotes

1 http://drive5.com/uparse
